# Widespread FUS mislocalization is a molecular hallmark of amyotrophic lateral sclerosis

**DOI:** 10.1093/brain/awz217

**Published:** 2019-08-01

**Authors:** Giulia E Tyzack, Raphaelle Luisier, Doaa M Taha, Jacob Neeves, Miha Modic, Jamie S Mitchell, Ione Meyer, Linda Greensmith, Jia Newcombe, Jernej Ule, Nicholas M Luscombe, Rickie Patani

**Affiliations:** 1 The Francis Crick Institute, 1 Midland Road, London, UK; 2 Department of Neuromuscular Diseases, UCL Queen Square Institute of Neurology, Queen Square, London, UK; 3 NeuroResource, Department of Neuroinflammation, UCL Queen Square Institute of Neurology, Queen Square, London, UK; 4 UCL Genetics Institute, University College London, Gower Street, London, UK; 5 Okinawa Institute of Science and Technology Graduate University, Okinawa 904–0495, Japan

**Keywords:** amyotrophic lateral sclerosis (ALS), fused in sarcoma FUS, RNA binding protein, intron retention

## Abstract

Mutations causing amyotrophic lateral sclerosis (ALS) clearly implicate ubiquitously expressed and predominantly nuclear RNA binding proteins, which form pathological cytoplasmic inclusions in this context. However, the possibility that wild-type RNA binding proteins mislocalize without necessarily becoming constituents of cytoplasmic inclusions themselves remains relatively unexplored. We hypothesized that nuclear-to-cytoplasmic mislocalization of the RNA binding protein fused in sarcoma (FUS), in an unaggregated state, may occur more widely in ALS than previously recognized. To address this hypothesis, we analysed motor neurons from a human ALS induced-pluripotent stem cell model caused by the VCP mutation. Additionally, we examined mouse transgenic models and post-mortem tissue from human sporadic ALS cases. We report nuclear-to-cytoplasmic mislocalization of FUS in both VCP-mutation related ALS and, crucially, in sporadic ALS spinal cord tissue from multiple cases. Furthermore, we provide evidence that FUS protein binds to an aberrantly retained intron within the SFPQ transcript, which is exported from the nucleus into the cytoplasm. Collectively, these data support a model for ALS pathogenesis whereby aberrant intron retention in SFPQ transcripts contributes to FUS mislocalization through their direct interaction and nuclear export. In summary, we report widespread mislocalization of the FUS protein in ALS and propose a putative underlying mechanism for this process.

## Teaser

ALS remains incurable due to an incomplete understanding of its molecular pathogenesis. Tyzack, Luisier *et al.* report that FUS protein, previously thought to mislocalise only in patients with *FUS* mutations, is also mislocalised in many cases of sporadic ALS. They further propose an underlying molecular mechanism for the mislocalisation.

**See Vidal and Atkin (doi:10.1093/brain/awz256) for a scientific commentary on this article.**

## Introduction

Amyotrophic lateral sclerosis (ALS) is a relentlessly progressive neurodegenerative condition, which remains incurable due to our incomplete understanding of the molecular pathogenesis. Genetic discoveries in ALS strongly implicate ubiquitously expressed regulators of RNA-processing (Taylor *et al.*, 2016). Pathologically, in 97% of cases, TDP-43 protein is mislocalized from the nucleus to the cytoplasm, where it aggregates (Neumann *et al.*, 2006). However, ALS-causing mutations in fused in sarcoma (FUS) and superoxide dismutase 1 (SOD1) conspicuously lack TDP-43 proteinopathy in the majority of cases (Mackenzie *et al.*, 2007). FUS aggregation is a recognized feature of FUS mutation-related ALS (Vance *et al.*, 2009). However, the concept that wild-type FUS nuclear-to-cytoplasmic mislocalization (rather than aggregation) might be a more widespread feature of other forms of ALS has not been systematically assessed to our knowledge. Indeed such mislocalization may have evaded detection thus far due to bias towards studying aggregates/inclusions rather than perturbed subcellular localization of unaggregated proteins alone. This possibility is strengthened by the facts that (i) impaired nuclear-cytoplasmic compartmentalization is increasingly recognized as a key feature of ALS (Boeynaems *et al.*, 2016); and (ii) FUS is known to shuttle between the nucleus and cytoplasm.

Here we systematically investigated FUS protein localization across a human induced pluripotent stem cell (iPSC) model, mouse transgenic models and human post-mortem tissue from multiple cases of sporadic ALS. We find that the nuclear-to-cytoplasmic mislocalization of FUS is a more widespread feature of ALS than previously recognized. Furthermore, we present evidence that supports a putative molecular mechanism for this mislocalization through interaction between FUS protein and the aberrant intron-retaining SFPQ transcript.

## Materials and methods

### Induced pluripotent stem cell culture and motor neuron differentiation

IPSCs were maintained using standard protocols. Motor neuron differentiation was carried out as described previously (Hall *et al.*, 2017; Luisier *et al.*, 2018). See Supplementary material for more detailed information. Details of iPSC lines are provided in Supplementary Table 1.

### Animals, transgenic models and tissue processing

All experiments were carried out following the guidelines of the UCL Institute of Neurology Genetic Manipulation and Ethic Committees and in accordance with the European Community Council Directive of November 24, 1986 (86/609/EEC). Animal experiments were undertaken under licence from the UK Home Office in accordance with the Animals (Scientific Procedures) Act 1986 (Amended Regulations 2012) and were approved by the Ethical Review Panel of the Institute of Neurology. The following transgenic mouse lines were used, and were analysed as different experimental groups: (i) female *SOD1^G93A^*mice [*B6SJL-Tg*(SOD1*G93A)*1Gur*/*J*, Jackson Laboratories], postnatal Day 93–95 (P93–95) (*n = *4 mice); (ii) male over-expressing mutant human VCP^A232E^ were generated by J. Paul Taylor *et al.*, St Jude Children’s Research Hospital, Memphis, TN, USA and are described in Custer *et al.* (2010) and bred to wild-type C57 Black 6 (*C57*/*B6*) background, symptomatic, 9 months old (*n = *3 mice); and (iii) wild-type *C56BL*/*6-SJL *mixed background (Jackson Laboratories) were used as control (*n = *4 mice). Mice were bred and maintained at the UCL Institute of Neurology in standard individually ventilated cages with up to three mice per cage, in a temperature and humidity controlled environment with a 12-h light/dark cycle and had access to drinking water and food *ad libitum.* Cages were checked daily to ensure animal welfare. Body weight was assessed regularly to ensure no weight loss. For tissue collection, animals were injected with terminal anaesthesia (pentobarbital sodium, Euthatal) and were transcardially perfused with 4% paraformaldehyde. The lumbar region of the spinal cord was removed and post-fixed with 4% paraformaldehyde and cryoprotected overnight with 30% sucrose; 10 or 20 μm serial transverse cryosections were cut for immunofluorescence staining.

### Human post-mortem tissue

Snap frozen tissue sections were obtained from lumbar spinal cords of eight healthy donors and 12 age and sex matched sporadic ALS patients (Supplementary Table 2). Death to snap-freezing delay times were also comparable between the groups [mean delay ± standard deviation (SD): 30.13 ± 12.87 and 27.75 ± 10.63 h, for control and sporadic ALS patients, respectively]. The spinal cord samples were obtained from the tissue bank NeuroResource, UCL Institute of Neurology, London, UK. Samples were donated to the tissue bank with written tissue donor informed consent following ethical review by the NHS NRES Committee London–Central and stored under a Research Sector Licence from the UK Human Tissue Authority (HTA). In this study, ALS cases were considered sporadic by the absence of any family history of motor neuron disease. Furthermore, all cases showed TDP-43 pathology when screened by NeuroResource, suggesting that they are not SOD1 mutants [as TDP-43 pathology is absent in SOD1-related ALS cases (Mackenzie *et al.*, 2007)].

### Immunolabelling, imaging and image analysis

For immunocytochemistry and immunohistochemistry, samples were blocked in 10% normal goat serum (NGS) or 10% normal donkey serum (NDS) as appropriate and permeabilized in 0.3% Triton™ X-100 [Sigma-Aldrich; in phosphate-buffered saline (PBS)] at room temperature for 1 h. Immunolabelling was performed with primary antibodies in NGS (5%) and Triton™ X-100 (0.1% in PBS) at 4°C overnight followed by species-specific secondary antibodies for 1 h at room temperature and DAPI nuclear counterstain (100 ng/ml) for 10 min at room temperature. For human post-mortem samples fixation and permeabilization in cold methanol (−20°C, 20 min) was performed before the immunostaining. Primary antibodies were diluted as follows: goat anti ChAT (Millipore, AB144P) 1:100; rabbit and mouse anti FUS (Abcam, ab84078 1:500; and Santa Cruz, sc-47711 1:100 respectively). Images were acquired using either a 710 Laser Scanning Confocal Microscope (Zeiss) or the Opera Phenix High-Content Screening System (Perkin Elmer). Images were acquired as confocal *z*-stacks with a *z*-step of 1 μm and processed to obtain a maximum intensity projection. For the analysis of nuclear/cytoplasmic ratio of FUS in iPSC-derived cells, images were analysed using the Columbus Image Analysis System (Perkin Elmer). The animal and post-mortem tissue sections were analysed using Fiji. Motor neurons were identified by choline acetyltransferase (ChAT) immunoreactivity, and the nuclear and cytoplasmic area were manually drawn based on DAPI and ChAT staining, respectively. For each cell, the average FUS immunoreactivity intensity in each region of interest was measured, background was subtracted, and the ratio between nuclear and cytoplasmic average intensity was calculated and used as the main experimental outcome.

### Cell fractionation, RNA extraction, reverse transcription and quantitative PCR

For the biochemical fractionation of iPSC-derived neural precursors the Ambion PARIS kit (Thermo Fisher Scientific) was used following manufacturer’s instructions. A cytosolic fraction was obtained by lysing the cultures for 10 min in ice-cold cell fractionation buffer. Nuclei were lysed in 8 M Urea Nuclear Lysis Buffer, containing 50 mM Tris-HCl (pH 8), 100 mM NaCl, 0.1% SDS, 1 mM DTT. Both lysis buffer contained 0.1 U/μl RiboLock RNase Inhibitor (Thermo Fisher Scientific). RNA was extracted from both fractions using the Promega Maxwell® RSC simplyRNA cells kit including DNase treatment, alongside the Maxwell® RSC instrument. Reverse transcription was performed using the RevertAid™ First Strand cDNA Synthesis Kit (Thermo Fisher Scientific) using 1 μg of RNA and random hexamers. Quantitative PCR was performed using the PowerUP™ SYBR® Green Master Mix (Thermo Fisher Scientific) and the the QuantStudio™ 6 Flex Real-Time PCR System (Applied Biosystems). Specific amplification was determined by melt curve analysis and agarose gel electrophoresis of the PCR products. Analysis of intron retaining transcripts was performed as previously described (Luisier *et al.*, 2018). Levels of intron retention (primer pair F2R2) were normalized over the expression level of each individual gene (primer pair F1R1). Primers used were as follows: SFPQ F1: GCCGAATGGGCTACATGGAT; SFPQ R1: TCAGTACG CATGTCACTTCCC; SFPQ F2: GTGGATCGACTCATTGGT GA; SFPQ R2: TTCCTCTAGGACCCTGTCCA.

### Single molecule fluorescence *in situ* hybridization

Based on Raj *et al.* (2008), iPSC-derived neural precursors (Day 7 after beginning of neural conversion) that were grown on Geltrex®-coated sterile 2-well µ-Slides (80286, Ibidi) were washed twice for 5 min with PBS, and fixed using 4% methanol-free formaldehyde (10321714, Thermo Fisher Scientific) for 10 min at room temperature. Following additional two wash steps, cells were permeabilized using 70% ethanol for 12 h at 4°C, and were washed twice again. Then preparations were incubated for 15 min with hybridization buffer prepared using 2× saline-sodium citrate (SSC) solution/10% deionized formamide (4610-OP, Calbiochem). Hybridization with Stellaris FISH probes was done in a total volume of 50 µl hybridization buffer containing 50 µg competitor tRNA from *Escherichia coli* (10109541001, Roche Diagnostics), 10% dextran sulphate (9011–18–1, VWR), 2 mg/ml UltraPure BSA (AM2616, Thermo Fisher Scientific), and 10 mM vanadyl-ribonucleoside complex (S1402S, New England Biolabs) with probes at final concentration of 1 ng/µl. Preparations were covered with parafilm and incubated at 37°C for 5 h, and afterwards washed twice with pre-warmed 2 × SCC/10% formamide for 30 min at 37°C. Finally the preparations were washed twice with PBS at room temperature, and then mounted using 10 µl ProLong® Gold Antifade Reagent containing DAPI (9071S, New England Biolabs). The slides were imaged when the mounting medium was fully cured >12 h.

Probes were designed using the Probe Designer software from Biosearch Technologies and were provided by same vendor. Probes included were designed against SFPQ intron conjugated to Quasar®570 (SMF-2037–1) and mature SFPQ conjugated to Quasar®670 (sequences of probes available upon request). Quantification of hybridization signal was performed using custom spot-intensity detection algorithm in DAPI segmented cells to separate nuclear and cytoplasmic signal.

### Data analysis of FUS protein subcellular localization

We used R and lme4 (Bates *et al.*, 2015) to perform linear mixed effects analysis of the relationship between FUS localization and VCP or SOD1 mutation, as well as with sporadic ALS, that accounts for idiosyncratic variation due to either animal or individual differences. As fixed effects, we either entered the mutation or the ALS disease variable into the model. As random effects, we had intercepts for either animals (SOD1, VCP) or patients and batches. Visual inspection of residual plots did not reveal any obvious deviations from homoscedasticity or normality. *P*-values were obtained by likelihood ratio tests of the full model with the effect in question against the model without the effect in question.

### Mapping of iCLIP data

Raw FUS individual-nucleotide resolution cross-linking and immunoprecipitation (iCLIP) data can be accessed at https://imaps.genialis.com/ (Attig *et al.*, 2018). Before alignment, two-stage adapter removal was performed using Cutadapt according to the ENCODE iCLIP standard operating procedure. A two-stage approach was also used for alignment. First, Bowtie2 was used to remove reads aligning to rRNA or tRNA. Then, STAR was used to align the remaining reads to GRCh38, with only uniquely mapping reads retained. PCR duplicates were collapsed based on the unique molecular identifiers and mapping locations. The nucleotide-resolution crosslink position was calculated as the coordinate immediately preceding the reverse transcription truncation event.

### Compliance with ethical standards

For human iPSC work, informed consent was obtained from all patients and healthy controls in this study. Experimental protocols were all carried out according to approved regulations and guidelines by UCLH’s National Hospital for Neurology and Neurosurgery and UCL Queen Square Institute of Neurology joint research ethics committee (09/0272). The human post-mortem spinal cord samples were obtained from the tissue bank NeuroResource, UCL Queen Square Institute of Neurology, London, UK. Samples were donated to the tissue bank with written tissue donor informed consent following ethical review by the NHS NRES Committee London–Central and stored under a Research Sector Licence from the UK Human Tissue Authority (HTA). All animal experiments described in this study were carried out under licence from the UK Home Office, and were approved by the Ethical Review Panel of the Institute of Neurology.

### Data availability

Data supporting the findings of this study are available from the corresponding author, upon reasonable request.

## Results

### Nuclear-to-cytoplasmic mislocalization of FUS in human and mouse VCP-mutant ALS models

**Figure 1:**
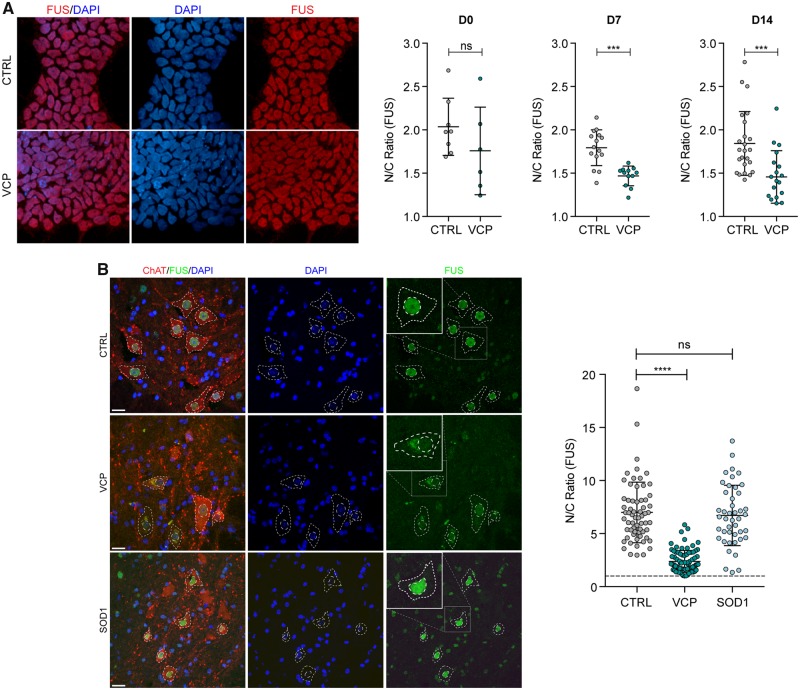
**Nuclear-to-cytoplasmic mislocalization of FUS in human and mouse VCP-mutant ALS models.** (**A**) Subcellular localization of FUS determined by immunocytochemistry in iPSCs (D0), neural precursors (NPCs; D7) and patterned motor neuron precursors (pMNs; D14). The ratio of the average intensity of the FUS immunolabelling in the nucleus (N) versus cytoplasm (C) was automatically determined in both CTRL and VCP mutant lines cells. Data shown are average N/C ratios (±SD) per field of view from four control and four VCP mutant lines. *P*-value from unpaired *t*-test with Welch’s correction. (**B**) Analysis of the subcellular localization of FUS in motor neurons in the ventral spinal cord of wild-type, *VCP^A232E^* and *SOD1^G93A^* mice. Motor neuron cytoplasm was identified by ChAT staining, nuclei were counterstained with DAPI. Images were acquired as confocal *z*-stacks using a Zeiss 710 confocal microscope with a *z*-step of 1 μm, processed to obtain a maximum intensity projection and analysed using Fiji. Motor neurons were identified by ChAT staining, and the nuclear and cytoplasmic area were manually drawn based on DAPI and ChAT staining, respectively. For each region of interest, the average FUS immunoreactivity intensity was measured, background was subtracted, and the ratio between nuclear and cytoplasmic average intensity was calculated. Data shown are nuclear/cytoplasmic (N/C) ratio (mean ± SD) per cell from four wild-type, four *SOD1^G93A^* and three *VCP^A232E^* mice. Scale bar = 20 μm. Linear mixed effects analysis of the relationship between FUS localization and either VCP or SOD1 mutation to account for idiosyncratic variation due to animal differences: SOD1 mutation does not affect FUS localization [2(1) = 0.6387, *P* = 0.4242], lowering it by about −0.8152 ± 1.1152 (standard errors), while VCP mutation does affect FUS localization [2(1) = 8.3145, *P* = 0.003933], lowering it by −4.2175 ± 0.9731 (standard errors).

Previously, we reported robust differentiation of human iPSCs into highly enriched (>85%) cultures of comprehensively validated and functionally characterized spinal cord motor neurons (Hall *et al.*, 2017; Maffioletti *et al.*, 2018). Using this approach, we have found clear molecular pathogenic phenotypes in a human iPSC model of VCP-related ALS (four clones from two patients with the mutations VCP^R155C^ and VCP^R191Q^) (Hall *et al.*, 2017; Luisier *et al.*, 2018). We harnessed this well-characterized ALS model to investigate the subcellular localization of FUS protein, which revealed a modest but statistically significant (*P* < 0.001) decrease in nuclear-to-cytoplasmic localization during motor neuron differentiation (Fig. 1A). We next sought to validate this finding *in vivo* by examining tissue sections from symptomatic transgenic VCP*^A232E^* mice. Additionally, we examined tissue from the SOD1*^G93A ^*mouse model as a comparator. Using linear mixed model analysis that accounts for inter-animal variation we showed that although FUS remained within the nucleus in the SOD1 mouse model [2(1) = 0.031, *P* = 0.955], nuclear-to-cytoplasmic mislocalization abounded in the VCP mouse model, with a reduction in FUS nuclear to cytoplasmic ratio of −4.0176 ± 1.1775 [2(1) = 8.164, *P* = 0.0042] (Fig. 1B).

### Nuclear-to-cytoplasmic mislocalization of FUS in human sporadic ALS

**Figure 2:**
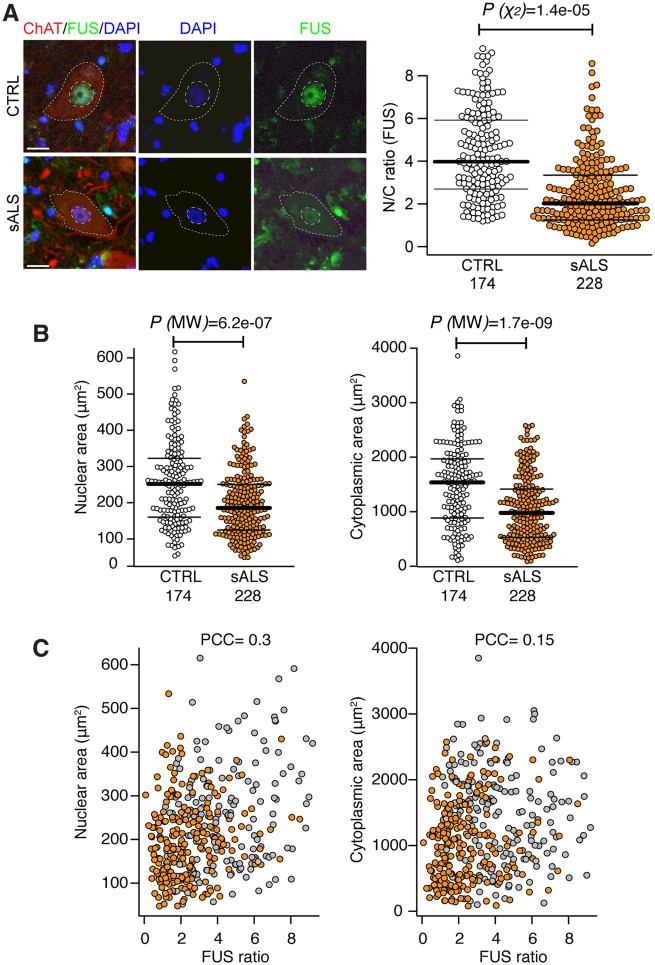
**Nuclear-to-cytoplasmic mislocalization of FUS in human sporadic ALS.** (**A**) Analysis of the subcellular localization of FUS in motor neurons in the ventral spinal cord of healthy controls (*n = *8) and patients with sporadic ALS (sALS) (*n = *12). Motor neuron cytoplasm was identified by ChAT immunolabelling, nuclei were counterstained with DAPI. N/C ratio of FUS immunoreactivity was measured as described in Fig. 1B. Only motor neurons with a visible nucleus were considered for the analysis. We used R and lme4 (Bates *et al.*, 2015) to perform a linear mixed effects analysis of the relationship between FUS localization and sALS disease. Scale bar = 20 μm. Data shown are N/C ratio (mean ± SD) per cell from at least eight cases per group. (**B**) Nuclear and cytoplasmic areas of motor neuron nuclei and cytoplasm in sALS (*n = *12) versus control (*n = *8). Data shown are mean ± SD. *P*-values from non-parametric Mann-Whitney’s test. (**C**) Single-cell measurement of nuclear (*right*) and cytoplasmic area (*left*) shows no correlation with FUS nuclear to cytoplasmic ratio as shown with scatter plots. PCC = Pearson correlation coefficient.

Having established that FUS is mislocalized in VCP-related ALS models, but not in SOD1, we next sought to address the generalizability of this finding across sporadic forms of ALS (which represent 90% of all cases). To this end, we examined post-mortem spinal cord tissue from 12 sporadic ALS cases and eight healthy controls (Fig. 2A). We found clear evidence of nuclear-to-cytoplasmic FUS mislocalization in these sporadic ALS cases, but in the absence of cytoplasmic FUS inclusions.

A reduction in motor neuronal soma size has been previously shown in an *in vitro* model of ALS which associates with increased apoptosis (Kiskinis *et al.*, 2014). We thus investigated whether changes in FUS subcellular localization in sporadic ALS samples accompany alterations in cell morphology. Using single-cell analysis of nuclear and cell size, we demonstrated significant reductions in both nuclear and cytoplasmic areas in sporadic ALS versus control motor neurons (Fig. 2B), which did not correlate with reduction in FUS nuclear-to-cytoplasmic localization in sALS cases (Fig. 2C).

### FUS binds to an aberrantly retained intron within the SFPQ transcript, which is exported from the nucleus

**Figure 3:**
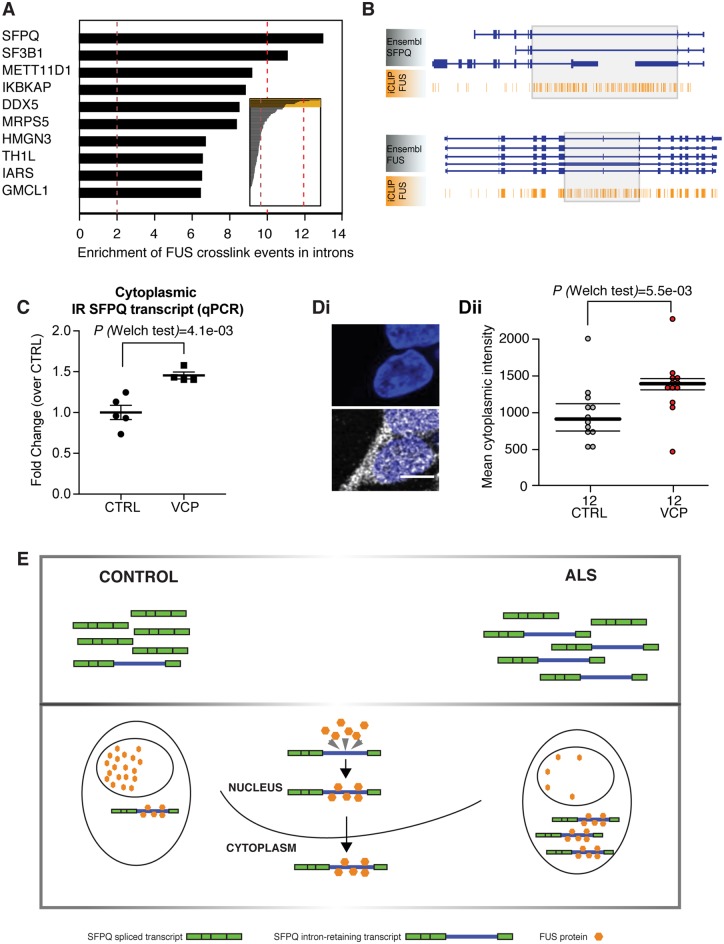
**FUS binds to an aberrantly retained intron within the SFPQ transcript, which is exported from the nucleus. **(**A**) Bar plots depicting the level of enrichment in FUS-binding to the retained intron compared with non-retained introns within the same gene. Analysis of the 167 previously reported as aberrantly retained introns in ALS. (**B**) Genome browser view of FUS iCLIP crosslinking events along the SFPQ and FUS transcript. Grey boxes highlight the location of retained introns. (**C**) Dot plot showing the levels of cytoplasmic intron retention (IR) SFPQ transcripts measured by qPCR. IR transcript levels were measured using an IR-specific primer pair and normalized over the expression levels of a constitutive SFPQ exon, as described previously (Luisier *et al.*, 2018). *n* = 5 control lines and *n* = 4 VCP lines, mean ± standard error of the mean (SEM) from two independent replicates per line, each analysed in technical duplicate. *P* = 0.0041 from Welch’s test. [**D**(**i**)] Single molecule fluorescence *in situ* hybridization (smFISH) showing SFPQ intron-retaining transcript in the cytoplasm. The *top* panel shows the nuclear staining only for context; the probe clearly detects intron-retaining transcripts in the cytoplasm. [**D**(**ii**)] Quantification and statistical analysis of smFISH performed on three control lines and three VCP mutant lines using Welch’s test. (**E**) Schematic diagram of proposed model. Cartoon summarizing the functional consequence of aberrant IR in SFPQ transcript. The 9 kb intron 9 in SFPQ is retained and exported to the cytoplasm. The FUS protein binds extensively to this retained intron. The FUS protein itself is abundantly relocalized from the nucleus to the cytoplasm in diverse ALS models (human iPSC and mouse transgenic), as well as post-mortem samples from patients with sporadic ALS.

Having found clear evidence that FUS nuclear-to-cytoplasmic mislocalization is more widespread in ALS than previously recognized, we sought to understand its molecular interplay with 167 aberrant intron retaining transcripts that we recently described in ALS (Luisier *et al.*, 2018). To this end we analysed iCLIP data, which allowed identification of RNA binding targets of the FUS protein. Using this approach, we found that FUS protein binds extensively to the aberrantly retained intron 9 within the SFPQ transcript (Fig. 3A and B), which we identified as the most significantly retained intron across diverse ALS mutations (Luisier *et al.*, 2018). We next confirmed that the SFPQ intron-retaining transcript is exported to the cytoplasm using nuclear-cytoplasmic cellular fractionation and qPCR (Fig. 3C), showing an increased proportion of SFPQ intron retaining transcript in the cytosol of VCP mutant cultures. We used single molecule RNA fluorescence *in situ *hybridization (smFISH) as orthogonal validation that this transcript is exported from the nucleus (Fig. 3D).

## Discussion

The core finding of our study is that the FUS protein is mislocalized from the nucleus to the cytoplasm in cases beyond FUS mutation-related ALS. Specifically, we find FUS mislocalization in VCP mutation-related ALS and, crucially, in sporadic ALS. Our findings have been carefully cross-validated in iPSC lines (four VCP mutant and four control iPSC lines), a mouse transgenic model (three VCP mutant and three control mice), and post-mortem tissue (12 sporadic ALS cases and eight control cases). The pervasive mislocalization of FUS has likely evaded detection thus far as FUS largely remains unaggregated in the cytoplasm, rather than forming part of the TDP-43 aggregates in sporadic ALS cases.

Our findings support a model whereby FUS mislocalization from the nucleus to the cytoplasm occurs in the majority of ALS cases, but it generally does not appear to aggregate in the cytoplasm. Nuclear loss of FUS protein may impair pre-mRNA splicing whilst the possibility of a cytosolic toxic gain of function is also noteworthy in light of recent studies (López-Erauskin *et al.*, 2018). Interestingly, TDP-43 aggregation is also observed in most familial and sporadic ALS cases, except from those caused by SOD1 mutations. Thus, FUS mislocalization appears to occur in a similar majority of cases to TDP-43 aggregation. Importantly, the mislocalized FUS is observed in mouse and iPSC models at early stages, before the onset of TDP-43 aggregates, indicating that the mislocalized unaggregated FUS might play a causative role early in the ALS disease process, perhaps by creating a more aggregation-prone cytoplasmic environment.

Our analysis of iCLIP data suggest that FUS binds avidly to the aberrantly retained intron of the SFPQ transcript in ALS. Cumulatively, our data are consistent with a working hypothesis that wild-type FUS might travel out of the nucleus when bound to the aberrantly retained intron 9 of the SFPQ transcript in ALS (Fig. 3E), though confirmation of such a mechanism will require detailed molecular follow-up work.

In our previous study, we showed SFPQ intron retention occurs in VCP, SOD1 and FUS mutant iPSC-motor neuron cultures and that this correlates with loss of nuclear SFPQ across different model systems (Luisier *et al.*, 2018). The current study, however, adds interesting complexity to this paradigm by suggesting that RNA-binding proteins sequestered by the SFPQ intron-retaining transcript might vary in a mutation-dependent manner. Indeed although SFPQ protein is sequestered by the SFPQ intron-retaining transcript across different mutations we examined, FUS is not statistically significantly sequestered and/or exported from the nucleus by this transcript in the context of SOD1 mutations. In addition to the evidence we provided in our previous work (Luisier *et al.*, 2018), mutant SOD1 has also been shown to bind RNA transcripts in other contexts, playing a role in their stabilization (Chen *et al.*, 2014; Lu *et al.*, 2007, 2009). Furthermore, SOD1 is known to form complexes with other ribonucleoproteins such as TIAR and HuR (Lu *et al.*, 2007, 2009). To further support the concept that mutant SOD1 can influence the localization (and therefore function) of other RNA binding proteins, a recent proteomic study found a nuclear shift in proteins involved in RNA transport and processing in SOD1 mutants compared to controls (Kim *et al.*, 2017). In aggregate, these studies raise the hypothesis that by altering RNA metabolism, mutations in SOD1 could lead to mutation-dependent changes in the molecular composition of ribonucleoprotein complexes. Specifically, mutant SOD1 might affect the interaction of specific RNA binding proteins, such as FUS, with their target RNAs. The absence of FUS mislocalization in this context, together with the lack of TDP-43 proteinopathy, seems to further substantiate a recognized pathological divergence between SOD1-ALS and other forms of familial and sporadic ALS.

In summary, we report a previously unrecognized widespread mislocalization (but not aggregation) of FUS in ALS, and propose a putative context-specific mechanism for this through its interaction with the ALS-related aberrantly retained intron 9 in SFPQ transcripts. These findings raise the prospect of targeting the nuclear-to-cytoplasmic mislocalization of unaggregated FUS as a putative therapeutic strategy in ALS.

## Supplementary Material

awz217_Supplementary_Data

## Abbreviations

ALS: amyotrophic lateral sclerosis
iCLIP: individual-nucleotide resolution cross-linking and immunoprecipitation
iPSC: induced pluripotent stem cell
