# Supplementary material for: Widespread FUS mislocalization is a molecular hallmark of amyotrophic lateral sclerosis
Source: Brain. 2019 Aug 1;142(9):2572–80. doi: 10.1093/brain/awz217 (PMC6735815; doi:10.1093/brain/awz217)
Supplement: awz217_Supplementary_Data [file awz217_supplementary_data.pdf]

Supplementary information

Supplementary methods

Derivation of Human Fibroblasts and iPSC

Dermal fibroblasts were cultured in OptiMEM +10% FCS medium. The following episomal plasmids were transfected for iPSC generation: pCXLE hOct4 shp53, pCXLE hSK, and pCXLE hUL (Addgene), as previously reported (Okita *et al.*, 2011). Details of the lines used in this study are provided in Table S1. Three of the control lines used (control 2 and control 3, and control 5) are commercially available and were purchased from Coriell (cat. number ND41866*C), ThermoFisher Scientific (cat. number A18945) and Cedars Sinai (Cat.number CS02iCTR-NTn4) respectively.

Cell culture and motor neuron differentiation

Induced PSCs were maintained on Geltrex (Life Technologies) with Essential 8 Medium media (Life Technologies), and passaged using EDTA (Life Technologies, 0.5mM). All cell cultures were maintained at 37°C and 5% carbon dioxide. For motor neuron (MN) differentiation, iPSCs were first differentiated to neuroepithelium by plating to 100% confluency in chemically defined medium consisting of DMEM/F12 Glutamax, Neurobasal, L-Glutamine, N2 supplement, non-essential amino acids, B27 supplement, β-mercaptoethanol (all from Life Technologies) and insulin (Sigma). Treatment with small molecules from day 0-7 was as follows: 1µM Dorsomorphin (Millipore), 2µM SB431542 (Tocris Bioscience), and 3.3µM CHIR99021 (Miltenyi Biotec). At day 8, cells patterned for 7 days with 0.5µM retinoic acid and 1µM Purmorphamine. At day 14 spinal cord MN precursors were treated with 0.1µM Purmorphamine for a further 4 days before being terminally differentiated for >10 days in 0.1 µM Compound E (Enzo Life Sciences) to promote cell cycle exit. Throughout the neural conversion and patterning phase (D0-18) the neuroepithelial layer was enzymatically dissociated twice (at D4-5 and D10-12) using dispase (GIBCO, 1 mg ml^{-1}). At relevant timepoints cells were harvested for RNA extraction or fixed in 4% paraformaldehyde for immunolabelling.

Supplementary table 1. Details of iPSC lines used in this study

iPSC line	Mutation present	Age of Donor	Age at disease onset	Sex of Donor
**CTRL 1**	None	78	N/A	Male
**CTRL 2**	None	64	N/A	Male
**CTRL 3**	None	Unknown	N/A	Female
**CTRL 4**	None	51	N/A	Female
**CTRL 5**	None	51	N/A	Male
**MUT 1**	VCP R155C	43	40	Female
**MUT 2**	VCP R155C	43	40	Female
**MUT 3**	VCP R191Q	42	36	Male
**MUT 4**	VCP R191Q	42	36	Male

Supplementary table 2. Post-mortem tissue case information.

Age	Gender	Category	Cause of death	Post mortem delay (hours)
71	M	Control	Burst aortic aneurysm.	25
68	F	Control	Colorectal metastatic cancer	23
68	M	Control	Heart disease	40
70	M	Control	Bronchopneumonia and heart failure	53
77	M	Control	Pulmonary fibrosis	32
80	F	Control	Pulmonary embolism	24
81	M	Control	Lung carcinoma; bilateral bronchopneumonia	10
67	F	Control	Bronchopneumonia	34
69	M	sALS	MND & stage 2 respiratory failure.	19
61	M	sALS	MND.	29
74	F	sALS	MND.	27
70	M	sALS	MND.	40
81	F	sALS	MND.	33
65	F	sALS	MND	30
70	F	sALS	MND	26
58	M	sALS	MND	49
69	M	sALS	MND	19
66	F	sALS	MND	15
77	F	sALS	Bronchopneumonia and MND	34
80	F	sALS	MND	12
